# Structural dynamics of Na^+^ and Ca^2+^ interactions with full-size mammalian NCX

**DOI:** 10.1038/s42003-024-06159-9

**Published:** 2024-04-16

**Authors:** Moshe Giladi, Lukáš Fojtík, Tali Strauss, Benny Da’adoosh, Reuben Hiller, Petr Man, Daniel Khananshvili

**Affiliations:** 1 Department of Physiology and Pharmacology, Faculty of Medicine, Tel-Aviv University, Tel Aviv, 69978 Israel; 2 Tel-Aviv Sourasky Medical Center, Tel Aviv, 6423906 Israel; 3 Division BioCeV, Institute of Microbiology of the Czech Academy of Sciences, Prumyslova, 595 252 50 Vestec, Prague, Czech Republic; 4 Department of Biochemistry, Faculty of Science, Charles University, 128 00 Prague, Czech Republic; 5 Blavatnik Center for Drug Discovery, Tel Aviv University, Tel Aviv, 69978 Israel

**Keywords:** Single-molecule biophysics, Ion channels, Membrane biophysics

## Abstract

Cytosolic Ca^2+^ and Na^+^ allosterically regulate Na^+^/Ca^2+^ exchanger (NCX) proteins to vary the NCX-mediated Ca^2+^ entry/exit rates in diverse cell types. To resolve the structure-based dynamic mechanisms underlying the ion-dependent allosteric regulation in mammalian NCXs, we analyze the apo, Ca^2+^, and Na^+^-bound species of the brain NCX1.4 variant using hydrogen-deuterium exchange mass spectrometry (HDX-MS) and molecular dynamics (MD) simulations. Ca^2+^ binding to the cytosolic regulatory domains (CBD1 and CBD2) rigidifies the intracellular regulatory loop (5L6) and promotes its interaction with the membrane domains. Either Na^+^ or Ca^2+^ stabilizes the intracellular portions of transmembrane helices TM3, TM4, TM9, TM10, and their connecting loops (3L4 and 9L10), thereby exposing previously unappreciated regulatory sites. Ca^2+^ or Na^+^ also rigidifies the palmitoylation domain (TMH2), and neighboring TM1/TM6 bundle, thereby uncovering a structural entity for modulating the ion transport rates. The present analysis provides new structure-dynamic clues underlying the regulatory diversity among tissue-specific NCX variants.

## Abstract (web summary)

Using HDX-MS and MD simulations, the authors demonstrate that Ca^2+^ and Na^+^ binding to specific domains rigidifies specific structural elements of the NCX protein, revealing regulatory modules and molecular mechanisms governing ion transport rates.

## Introduction

The cell-membrane bound Na^+^/Ca^2+^ exchangers (NCXs) represent a large group of highly conserved proteins that shape Ca^2+^ signaling/homeostasis in different cell types across the kingdoms of life^1–4^ .In mammals, three isoforms (NCX1-3) and their splice variants are expressed in a tissue-specific manner to match cell-specific prerequisites under ever-changing physiological conditions^5–8^. Physiologically, NCX can mediate either the forward (Ca^2+^-exit) or reverse (Ca^2+^-entry) mode of ion exchange in both excitable and non-excitable cells by facilitating the consecutive counter-transport of 3Na^+^ ions for 1Ca^2+^ ion across the cell membrane^2,4,9^. In addition to the electrochemical driving force of membrane potential and the ionic gradient, the ion-exchange rates of mammalian NCXs are allosterically regulated by ionic (Ca^2+^, Na^+^, and H^+^) and metabolic (ATP, PIP_2_, and lipids, among others) ligands^4–9^. Disease-related alterations in the NCX expression levels or regulation can considerably contribute to many maladies, although the long-wanted pharmacological targeting of NCX isoforms/splice variants remains challenging^4,7–9^.

**Fig. 1:**
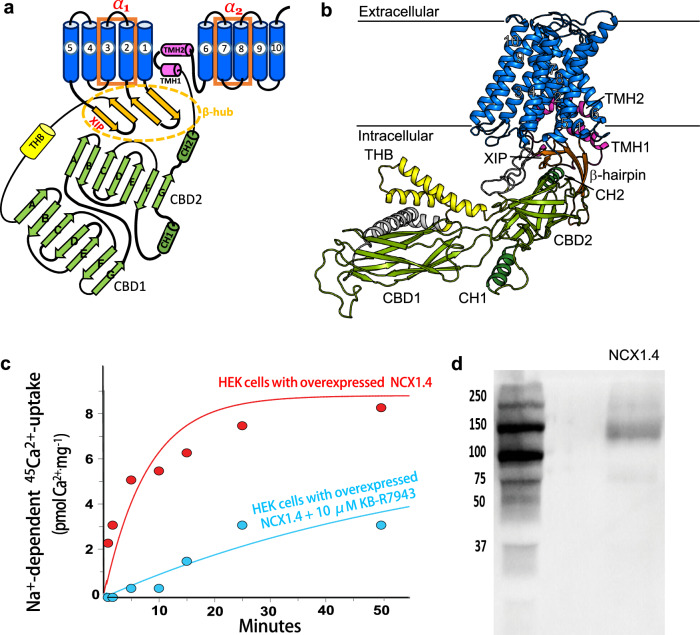
Overall structure and purification of NCX1.4. **a** Topological model of NCX1.4, based on the cryo-EM structure of NCX1.1. The region connecting TM5 and CBD1 was not resolved in this structure. **b** The structural model of NCX1.4, generated using AlphaFold and the cryo-EM structure of NCX1.1 as described in the Methods section and colored according to the structural elements in (**a**). The structure is in the side view, where the horizontal lines represent the membrane plane and the cytoplasmic part points downward. **c** The Na^+^/Ca^2+^ exchange activity was measured in HEK293F cells overexpressing NCX1.4 in the absence (red) or presence (cyan) of the NCX inhibitor KB-R7943. **d** SDS-PAGE analysis of purified NCX1.4.

Previous structural studies of prokaryotic NCX (in the outward-facing conformation) revealed a conserved topology of ten transmembrane helices (TM1-10) that form two inversely oriented hubs (TM1-5 and TM6-10) (Fig. 1a, b)^10,11^. Each hub contains a highly conserved α-repeat (α_1_, TM2/TM3; α_2_, TM7/TM8)^10,11^, where four binding sites (S_ext_, S_mid_, S_int_, and S_Ca_) alternatively bind either 3Na^+^ (at S_ext_, S_int_ and S_Ca_) or 1Ca^2+^ (at S_Ca_) during the transport cycle^10–15^. The binding of 3Na^+^ or 1Ca^2+^ to the membrane-embedded transport domains initiates alternating access of the ion-binding pocket at opposite sides of the membrane, although it remains unclear how the sliding of the TM1/TM6 helices on the protein surface can promote the swapping of inward-facing (IF) and outward-facing (OF) conformational states^10–17^.

In contrast to prokaryotic NCX, which encompasses a short loop (16–32 residues) between TM5 and TM6 (5L6)^10,11^, mammalian NCXs contain a huge (~520 residues) cytosolic 5L6 which plays a crucial role in allosteric regulation^1–3^. This regulatory loop includes several structural elements, including (from N- to C-terminus) the auto-inhibitory sequence (XIP)^18,19^, a two-helix bundle (THB) module^20^, regulatory Ca^2+^ binding domains 1 (CBD1)^21–23^, and 2 (CBD2)^21,24^, and a short palmitoylation helix (TMH2)^25–27^. Previous structure-functional and mutational studies have established three major modes of ion-dependent allosteric regulation involving the regulatory loop: Ca^2+^-dependent activation, Na^+^-induced inactivation, and Ca^2+^-dependent alleviation of Na^+^-induced inactivation^28–32^. Specifically, cytosolic Ca^2+^ binding to high-affinity sites of CBD1 activates the ion-transport rates (up to 25-fold), whereas Ca^2+^ binding to CBD2 alleviates Na^+^-induced inhibition^23,24,33^. Notably, in contrast to NCX1 and NCX3, the NCX2 isoform lacks Na^+^-induced inactivation^34^. To date, the location and structural identity of the Na^+^ binding site involved in allosteric regulation of mammalian NCXs remain unknown.

Previously, purified preparations of the two-domain CBD1-CBD2 (CBD12) tandem (derived from different isoforms/splice variants of mammalian NCX) were analyzed using biophysical approaches to elucidate the Ca^2+^-induced conformational changes involved in NCX regulation^22,35–45^. These studies have established a common mechanism underlying the Ca^2+^-dependent activation of NCXs, according to which, the high-affinity Ca^2+^ binding to CBD1 rigidifies the interdomain movements of CBDs, resulting in an elongated conformation of CBD12^45^. Notably, the exon composition within the splicing segment (exclusively located at CBD2) dictates the number of Ca^2+^ binding sites at CBD2 (from 0 to 3) and thus, predefines the ability of a given NCX variant to perform Ca^2+^-dependent alleviation of Na^+^-induced inactivation^4,9,22,40^. This paradigm is consistent with functional studies using full-size NCX variants demonstrating that Ca^2+^ cannot alleviate the Na^+^-induced inactivation in NCX1.3 or NCX3.1 (due to the lack of Ca^2+^ binding to CBD2). In contrast, the NCX1.1, NCX1.4, and NCX3.2 variants undergo Ca^2+^-dependent alleviation of Na^+^-induced inhibition (due to the Ca^2+^ binding to CBD2)^46–48^. Despite these findings, the structure-dynamic basis underlying the allosteric communications between the regulatory and transport domains has remained unresolved, given the predicted long distance (50–80 Å) between the regulatory and membrane-embedded transport domains.

Recently, the cryo-EM structures of antibody-bound cardiac NCX1.1^49^ and the kidney NCX1.3^50^ with bound inhibitor (SEA-0400) were resolved in inward-facing conformation, thereby providing breakthrough information on the 3D folding of mammalian NCX. The NCX1.1 structures were obtained in the presence of saturating Na^+^ and either residual (“inactivated”) or high Ca^2+^ (“activated”) concentrations^49^, whereas the NCX1.3 structure was resolved at high Ca^2+^ concentrations^50^. Notably, in both “activated” and “inactivated” structures of NCX1.1 the Ca^2+^ sites at CBD1 are occupied, consistent with previously described submicromolar affinity of Ca^2+^ binding sites at CBD1^21,23,35,37^. The NCX1.1 and NCX1.3 structures revealed a tightly packed four-stranded β-sheet structure (β-hub) at the interface between the cytosolic 5L6 loop and transmembrane region (Fig. 1a, b)^49,50^. The β1 and β2 strands of the β-hub come from the cytoplasmic loop between TM1 and TM2 (1L2), forming a curved β-hairpin extending into the cytosol. The β3 and β4 strands arise from the C-terminus of TM5, including the auto-inhibitory XIP sequence, forming a twisted antiparallel β-sheet that runs parallel to the internal membrane surface and anchors the hub to the TM domains via hydrophobic interactions. The cryo-EM structure of NCX1.1 depicts an α-helix (termed CH2) at the C-terminus of CBD2, which is partially cuffed by the β-hub in the inactivated state. This allows the formation of an inactivation assembly by the simultaneous interactions of the β-hub with the regulatory and transmembrane domains (Fig. 1b)^49^. Conversely, in the activated state, Ca^2+^ binding to CBD2 was suggested to destabilize the inactivation assembly, consequently separating the regulatory 5L6 loop from the transmembrane domains.

The recently derived cryo-EM structures of NCX1.1^49^ and NCX1.3^50^ open new opportunities for elucidating the mechanisms of Ca^2+^- and Na^+^-dependent allosteric regulation, although despite this progress, fundamental questions regarding the mechanisms underlying allosteric regulation remain open. For example, the regulatory Na^+^ binding site was not identified. Moreover, both structures were determined in the presence of bound Ca^2+^ at CBD1, which is known to be the primary Ca^2+^ sensor for mammalian NCX activation^21,23,35,38^; therefore, a fully inactivated state is missing. Furthermore, the region linking between TM5 and CBD1 (including the THB entity) in NCX1.1 and NCX1.3 was not resolved by the cryo-EM analysis, thereby suggesting that this region is highly dynamic^49,50^. Finally, the dissociation of the inactivation module resulted in poor resolution at the transmembrane domain since highly mobile structural elements on the cytosolic 5L6 loop might preclude high-resolution structural insights into the activated state^49^.

Since ion transport by NCX is an inherently dynamic process, we sought to investigate the structural dynamics of Ca^2+^- and Na^+^ interactions with mammalian NCX and resolve the allosteric interactions between the ion-transport and regulatory domains in full-size mammalian NCX1. To initiate this project, we developed experimental procedures for the overexpression and purification of functionally active NCX1.4 (a brain splice variant) in milligram quantities. These samples were analyzed for their Ca^2+^- or Na^+^-induced effects on the backbone dynamics using hydrogen-deuterium exchange mass spectrometry (HDX-MS) since this approach provides unique opportunities for detecting long-range allosteric interactions in large proteins^51–54^. As a complementary approach, we explored the established protocols of molecular dynamics (MD) simulations, aiming to detect specific structure-dynamic changes associated with Ca^2+^-mediated long-range allosteric regulation in full-size NCX1.4^55^. Our results revealed that in full-size NCX1.4, Ca^2+^ (but not Na^+^) effectively rigidifies the backbone of both CBDs and constrains their conformational landscape. In contrast, both Na^+^ or Ca^2+^ stabilize the intracellular portions of TM3, TM4, TM9, TM10, and their connecting loops (3L4 and 9L10), exposing a putative site for allosteric Na^+^ binding. Based on our findings, we also suggest that TMH2 interactions with the sliding bundle (TM1/TM6), as well as the Ca^2+^-related interactions of THB with membrane entities, may affect the dynamic features of the ion-transporting domains and thus, the transport rates in mammalian (but not in prokaryotic) NCXs.

## Results

### Expression and purification of functional full-size NCX1.4

To perform HDX-MS analyses of apo and ion-bound NCX1.4, we developed specific experimental protocols for its expression in transiently transfected HEK293F cells in suspension (Fig. 1c, d). To increase the expression levels of the NCX1.4 protein in the HEK293F cells, a codon-optimized,10xHis-tagged construct was used for overexpression and purification of the NCX1.4. The 40 kDa linear polyethyleneimine (PEI) was used for transient transfection, as much lower protein expression levels of NCX1.4 were obtained with 25 kDa PEI. Optimization tests have shown that a 1:3 ratio of DNA/PEI gives the maximal efficiency (30–50%) for the expression of the NCX1.4 protein in HEK293F cell suspension. The time-dependent tests of transient overexpression have shown that the highest levels of the NCX1.4 protein expression are achieved 8–12 h after transfection. To evaluate the functional activity of the overexpressed protein, we assayed NCX-mediated ion fluxes by measuring the Na^+^-dependent ^45^Ca^2+^-uptake and compared it to the mock-transfected HEK293F cells. This test demonstrates that NCX1.4 is a functionally active protein capable of mediating Na^+^/Ca^2+^ exchange (Fig. 1c). Moreover, adding 10 µM KB-R7943 (a well-established NCX-blocker) suppresses the Na^+^-dependent ^45^Ca^2+^-uptake in HEK-293F cells expressing NCX1.4 (Fig. 1c).

The NCX1.4 protein was solubilized from the isolated cell membranes of 20–25 gr HEK293F cells. The detergent concentrations were gradually decreased during the purification on the TALON column, where the detergent concentrations were kept 2–4 times higher than the CMC of DDM (0.12–0.17 mM) before loading the protein on the gel-filtration column. Under these conditions, NCX1.4 was eluted in a monodispersed peak from the gel filtration column in a buffer containing 0.25 mM (~2 CMC) DDM. 10% glycerol was included in the “storage buffer” to avoid the damaging effects of freezing-thawing. The yield of NCX1.4 preparations (at a purity of >90%) was in the range of 0.3–0.5 mg of protein per 1 L of HEK293F cell suspension (Fig. 1d). The purified NCX1.4 preparations were reconstituted into liposomes to measure Na^+^-dependent ^45^Ca^2+^-uptake (see Materials and Methods).

### HDX-MS analysis of detergent-solubilized apo full-size NCX1.4

The HDX-MS procedures that we have previously explored for prokaryotic NCX_Mj were further modified for the present analysis of full-size NCX1.4^14,15^. Owing to the inherent challenges in acquiring high sequence coverage for membrane proteins, stemming mainly from steric hindrance by detergent interactions with hydrophobic regions and sequence-dependent resistance to different digestive enzymes, we have carefully optimized the proteolytic conditions. First, we evaluated the influence of detergent concentration in the buffer. Not surprisingly, higher, or uncontrolled detergent concentration (when using centrifugal concentrators) led to overloading of the reversed-phase column capacity, poor separation, and low sequence coverage. Hence strict control of the detergent amount must be maintained, and no protein concentration should be utilized after the last purification step. Second, based on our previous experience with other membrane proteins, we utilized pure acid-based quench conditions and the addition of detergent. The best results were acquired when pure phosphoric acid supplemented with 0.25 mM (~1.5 CMC) DDM was used. Third, we fine-tuned the digestion conditions by testing various protease columns (pepsin, nepenthesin-2) and their combinations as well as the digestion temperature. Here, co-immobilized pepsin-nepenthesin-2 operated at 21 °C led to the highest sequence coverage and optimal peptide length. Our HDX workflow always contained an additional 20 s replicate/control test after running the entire HDX experiment. As the level of deuteration was the same as for the initial 20 s time points, protein degradation, unfolding, or other unwanted events can be ruled out with high confidence.

**Fig. 2:**
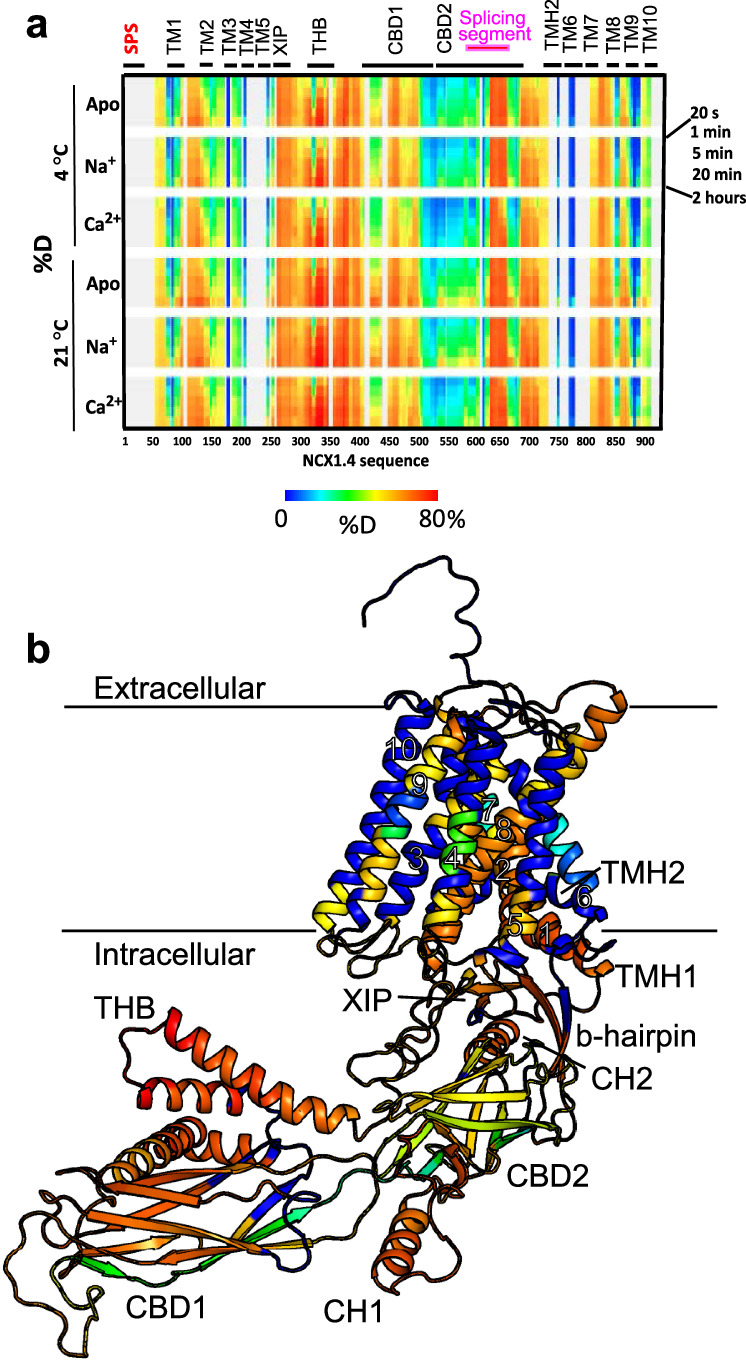
HDX-MS analysis of the apo, Ca^2^^+^-, and Na^+^-bound states of NCX1.4. **a** Deuteration levels at the indicated time points were obtained at 4 °C (upper panels) or 21 °C (lower panels). The apo (150 mM Choline-Cl), Na^+^ (100 mM NaCl + 50 mM Choline-Cl), and Ca^2+^ (150 mM Choline-Cl + 5 mM CaCl_2_) samples contained 20 mM Tris-HCl pH 7.5, 0.5 mM EGTA and 0.25 mM DDM (for more details see Materials and Methods). The highest and lowest exchange levels are denoted in red and blue, respectively. Structurally and functionally relevant elements are denoted above the heat maps created by MSTools. **b** Cartoon representation of NCX1.4 (in the apo state) is color-coded according to the deuterium uptake following 2 h of incubation in D_2_O at 21 °C. The structure represents an inside view, where the horizontal lines represent the membrane plane and the cytoplasmic part points downward. In all HDX-MS experiments, five different time points were taken (20 s, 1 min, 5 min, 20 min, and 2 h). The 20 s and 5 min timepoints were taken in triplicate and HDX-MS analyses were performed on two different batches of purified NCX1.4. To capture faster dynamic as well as poorly accessible regions, two HDX sets were tested at different temperatures (each set from a different batch). As can be seen in Supplementary Fig. 2, after accounting for the temperature effect on the intrinsic exchange rate, the datasets show a very high similarity.

Within the analysis of LC-MS/MS data, we also searched for possible post-translational modifications. We enabled Cys palmitoylation and Ser/Thr phosphorylation in the MASCOT search and searched manually for N-glycosylation via glycan-related oxonium ions. The C735 (C703, if the signal peptide sequence of 32 residues at the N-terminus is not accounted for) is reported to be acylated, but we failed to cover this region in the HDX data. However, the LC-MS/MS-based identifications provided unmodified peptides covering this region. Thus, it is possible that our preparation of purified NCX1.4 is either non-palmitoylated or this post-translational modification is only partial. Interestingly, we repeatedly found partial phosphorylation on Thr 284. The exact site should be taken with caution as it is based on CID data only and thus, this partial modification may be present in the region 280–290. Nevertheless, the phosphorylated peptide signals were weak and thus did not provide HDX data. Our search for the N-glycosylation failed to detect this modification despite the successful sequence coverage of most locations assigned to the putative N-glycosylation sites. Only the site Asn 41 repeatedly escaped our identification attempts and thus any conclusion about its glycan occupancy is not possible. Based on these observations, no modifications were considered in the final interpretation of the HDX data. Finally, no peptides corresponding to the signal peptide sequence (SPS, residues 1–32) were identified, suggesting that it undergoes cleavage during the post-translational processing of NCX1.4. Overall, the experimentally obtained sequence coverage is 74% and 77% with and without the SPS, respectively (Fig. 2, Supplementary Fig. 1). It should also be noted that this coverage is achieved by peptides providing the HDX data, whereas the actual coverage arising from LC-MS/MS identifications was usually exceedingly higher, reaching 85%.

The HDX profiles of apo NCX1.4 were obtained in a buffer composed of 20 mM Tris-Cl, pH 7.5, 150 mM choline-Cl, 0.5 mM EDTA, and 0.25 mM DDM at two different temperatures (4 °C or 21 °C). These two temperatures were selected to capture well-resolved HDX kinetics for both poorly structured/highly dynamic regions as well as highly folded and/or solvent-inaccessible parts. Overall, considering the change in the chemical exchange rate, the experimentally obtained heat maps of apo NCX1.4 are comparable at 4 °C or 21 °C, suggesting that the folding/unfolding equilibrium of the ligand-free protein is unaffected at this temperature range (Fig. 2a, Supplementary Fig. 2). This observation is interesting since both the prokaryotic and mammalian NCXs are sensitive to varying temperatures^12,56^. It seems that the HDX-MS technique cannot detect the temperature-dependent conformational changes in mammalian NCXs since they own to high turnover rates (10^3^–10^4 ^s^−1^) – this could be also the major reason, why the occupation of NCX1.4 transport sites by Na^+^ or Ca^2+^ was not detected in the present study. In contrast to NCX-1.4, the HDX-MS analysis has detected the binding of Na^+^ or Ca^2+^ to the transport sites of prokaryotic NCX_Mj, presumably because the turnover rates of NCX_Mj (0.5 s^−1^) are several orders of magnitude slower than the turnover rates of mammalian NCXs^12,57–59^.

To gain structural insights, we modeled the canine NCX1.4 according to the cryo-EM structure of human NCX1.1 to project the experimentally obtained HDX-MS data. Importantly, the deuterium uptake profiles (ranging between 0 and 80%) characteristically differ at specific regions, consistent with the cryo-EM structures of NCX1.1^49^ and NCX1.3^50^ and the derived structural model of full-size NCX1.4 (Fig. 2b). Specifically, the transmembrane helices not exposed to the bulk solution exhibit low HDX. In contrast, the transmembrane helices involved in ion transport (TM2, TM3, TM7, and TM8) exhibit higher deuterium uptake. These data underscore the exposure of the ion-transporting helices to the bulk phase facing the ion passageway entities, as expected for a natively folded NCX protein. Moreover, CBD1, known to undergo partial unfolding in the apo state, exhibits a markedly higher deuterium uptake compared with CBD2, which maintains its structural integrity in the absence of Ca^2+^^21,24^. Intriguingly, the inactivation module, consisting of CH2, the β-hub, and the cytosolic portions of TM1 and TM2, exhibits relatively high HDX despite the lack of bound Ca^2+^ at CBD2, suggesting that it is dynamic in the apo state. Altogether, the deuterium uptake profiles of apo NCX1.4 support the notion that our preparation of purified NCX1.4 is well-folded and suitable for studying the Ca^2+^- and Na^+^-induced effects on the backbone dynamics using HDX-MS.

### Ca^2+^ strongly rigidifies both CBDs in full-size NCX1.4

**Fig. 3:**
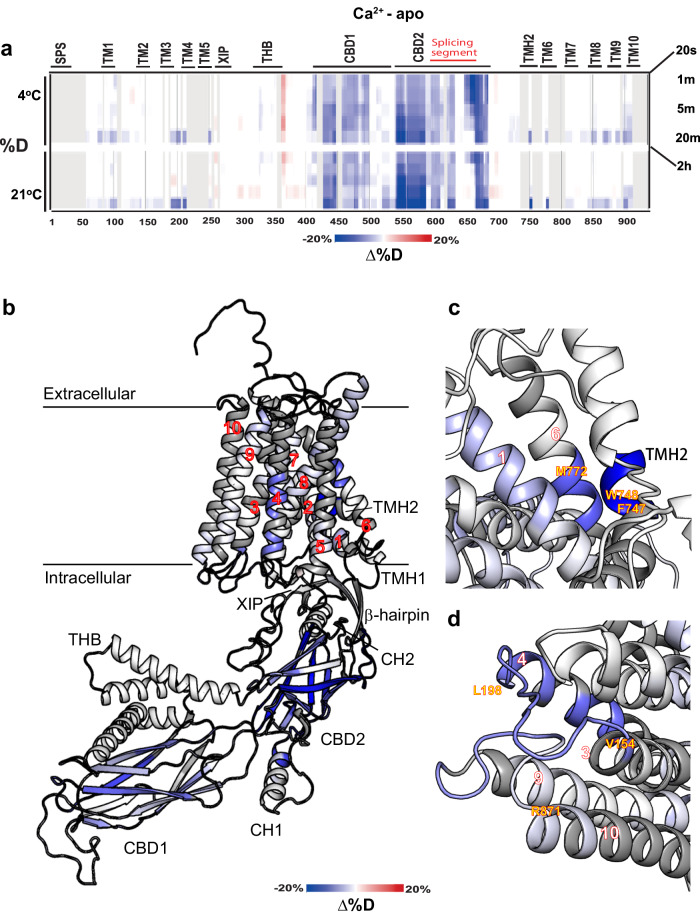
Ca^2+^-induced alterations in deuterium uptake. **a** Differential deuteration levels at the indicated time points obtained at 4 °C (lower panels) or 21 °C (upper panels) were calculated by subtracting the HDX level of the apo state from the Ca^2+^-bound state. Blue and red regions denote reduced and increased deuterium uptake, respectively, in the Ca^2+^-bound state. Structurally and functionally relevant elements are denoted above the heat maps. **b** Cartoon representation of NCX1.4 color-coded according to the differential deuterium uptake following 2 h of incubation in D_2_O. Close-up views of the TMH2-TM1-TM6 (**c**) and TM3-3L4-TM4/TM9-9L10-TM10 (**d**) clusters.

**Fig. 4:**
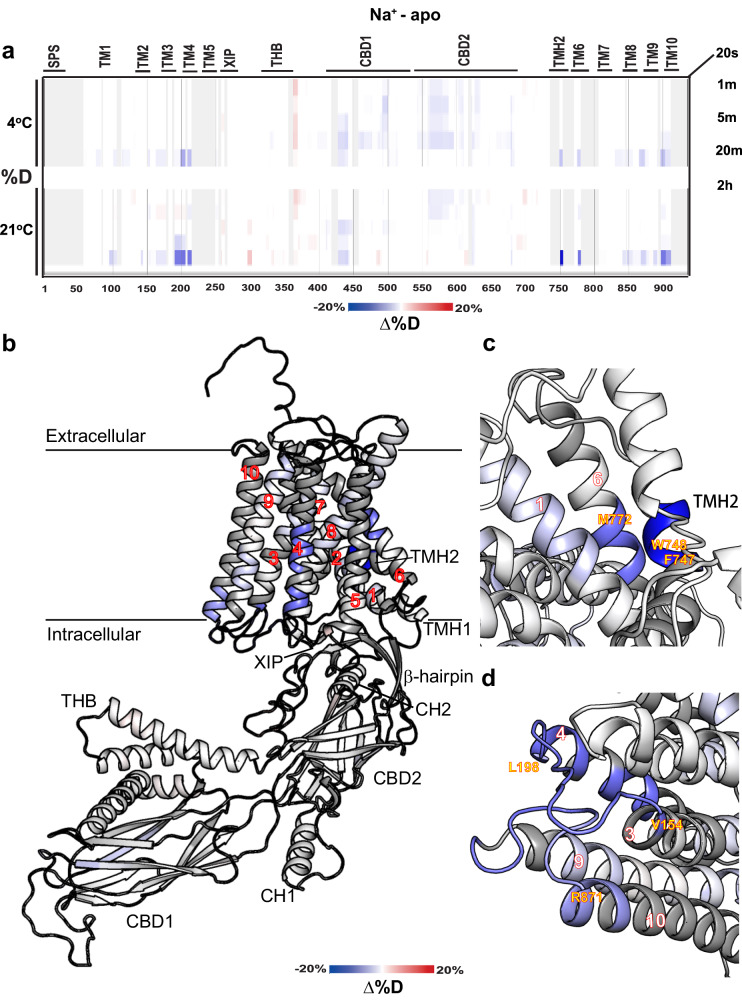
Na^+^-induced alterations in deuterium uptake. **a** Differential deuteration levels at the indicated time points obtained at 4 °C (lower panels) or 21 °C (upper panels) were calculated by subtracting the HDX level of the apo state from the Na^+^-bound state. Blue and red regions denote reduced and increased deuterium uptake, respectively, in the Na^+^-bound state. Structurally and functionally relevant elements are denoted above the heat maps. **b** Cartoon representation of NCX1.4 color-coded according to the differential deuterium uptake following 2 h of incubation in D_2_O. Close-up views of the TMH2-TM1-TM6 (**c**) and TM3-3L4-TM4/TM9-9L10-TM10 (**d**) clusters.

The Ca^2+^-dependent effects on the backbone dynamics of purified NCX1.4 were detected by subtracting the deuterium uptake of apo NCX1.4 (obtained in a buffer composed of 20 mM Tris-Cl, pH7.4, 150 mM choline-Cl, 0.5 mM EDTA and 0.25 mM DDM) from that of the Ca^2+^-bound state, obtained at saturating Ca^2+^ concentrations (in a buffer composed of 20 mM Tris-Cl, pH 7.5, 150 mM choline-Cl, 0.5 mM Ca^2+^, and 0.25 mM DDM) (Fig. 3a). Projecting the HDX profile differences onto the model of NCX1.4 reveals that overall, Ca^2+^ reduces the deuterium uptake (either at 4 °C or 21°) at specific locations of the cytosolic and membrane domains (Fig. 3b). As expected, Ca^2+^ reduces the deuterium uptake throughout CBD1 and CBD2, thereby representing the Ca^2+^-dependent rigidification of both CBDs (Fig. 3a, b). Notably, under comparable experimental conditions, high concentrations of Na^+^ (20 mM Tris-Cl, pH 7.5, 50 mM choline-Cl, 100 mM NaCl, 0.5 mM EDTA) do not affect the deuterium uptake at the CBDs (Fig. 4a, b), suggesting that Na^+^ does not interact with the Ca^2+^-binding sites of the CBDs in full-size NCX1.4. These HDX-MS data are consistent with a previous patch-clamp analysis of full-size NCX1.1^33^ and ^45^Ca^2+^ binding assays using isolated CBDs preparations, demonstrating that Na^+^ and Ca^2+^ do not compete for common binding sites^33,38^. Moreover, the ion-coordinating ligation centers of the CBDs are compatible with Ca^2+^ but not Na^+^ binding. Although one cannot exclude “nonselective” electrostatic interactions of Na^+^ with CBDs, these interactions were not shown to be of physiological significance.

An important observation is that Ca^2+^ rigidifies the tip of the α-helical region (CH1) located on the FG-loop, adjacent to the alternative splicing segment at CBD2 (Fig. 3b). This reflects the Ca^2+^-induced effect on interdomain movements in full-size NCX1.4, thereby representing the dynamic coupling of regulatory domains. Importantly, the HDX-MS analysis of isolated CBD12 variants revealed a very similar effect, where the strength and extent of Ca^2+^-induced rigidification is an exon-dependent event, which dramatically differs among NCX variants^4,39,43,44^. Intriguingly, there is no indication of a continual rigidification signal that connects CBDs with the transmembrane segments (Fig. 3b). This is consistent with previous findings suggesting that the Ca^2+^-driven dynamic coupling of CBD movements accounts for allosteric activation^4,8,35,45^. Moreover, the Ca^2+^-dependent slight stabilization of the N-terminal portion of CH2 (which is next to the C-terminus of CBD2) may contribute to the dynamic coupling of CBDs in the absence of any long-range backbone rigidification that spans from the cytosolic to the transmembrane domains. Notably, the HDX-MS analysis has detected only the Ca^2+^-dependent rigidification signals in full-size NCX1.4 (Fig. 3). This is difficult to reconcile with the previous proposal suggesting that the Ca^2+^-dependent destabilization of CBD2 can increase the conformational flexibility of nearby structural elements (located at the interface of the cytosolic and membranal entities) as well as of the downstream transmembrane helices^49^.

### Ca^2+^ and Na^+^ similarly rigidify distinct transmembrane clusters in full-size NCX1.4

In contrast with the CBDs, saturating concentrations of either Na^+^ or Ca^2+^ induce remarkably similar changes in deuterium uptake at distinct segments of the transmembrane domain and TMH2 (Figs. 3 and 4). Although subtle, ∆HDX signals were observed at specific segments of the ion-transporting helices TM2, TM3, TM7, and TM8 for Ca^2+^ (Fig. 3a, b, d) and at TM2, TM3, and TM8 for Na^+^ (Fig. 4a, b, d), consistent with the established coordination at the ion-transport sites^10,11,13^. The subtle change in HDX can be explained by the high turnover rates of NCX1.4 (~2500 s^−1^), resulting in exposure of ion binding sites to the bulk phase even in the presence of transported ions^57–59^.

An unexpected finding is that either Ca^2+^ (Fig. 3a) or Na^+^ (Fig. 4a) induces a robust decrease in deuterium uptake at two membrane-associated clusters of full-size NCX1.4 that are not directly involved in the ion passageway. The first cluster includes the interface between TMH2 (V713-F721), TM1 (I59-E69), and TM6 (L739-L745), where the location and strength of Ca^2+^ (Fig. 3b, c) or Na^+^ (Fig. 4b, c) induced effects are remarkably similar. At this end, it is unclear whether the observed rigidification of the TMH2-TM1-TM6 cluster represents direct Na^+^ or Ca^2+^ binding or distant allosteric interactions, instigated by Na^+^ or Ca^2+^ interaction with the transport sites. The underlying mechanisms may have a primary physiological relevance since the interaction of TMH2 (containing the palmitoylation site^25–27^) with the TM1/TM6 bundle (controlling the alternating access of the ion-binding pocket^10–17^) can modulate the conformational transitions of ion-transporting helices (TM2, TM3, TM7, and TM8) to vary ion transport rates. Since the TMH2 domain is absent in prokaryotic NCXs, the TMH2-TM1-TM6 cluster may contribute (at least partially) to 10^3^−10^4^-fold differences in the ion-transport rates between the mammalian and prokaryotic NCXs^12,57–59^.

In addition, we found that Ca^2+^ (Fig. 3d) or Na^+^ (Fig. 4d) rigidifies the backbone of TM3-3L4-TM4 (Y152-A179) and TM9-9L10-TM10 (Y857-L876) segments. Although both Na^+^ and Ca^2+^ have nearly identical effects on the 3L4 loop (C151-R167), at the 9L10 loop, Na^+^ has a more diffuse rigidifying effect (Y857-T878) (Fig. 4d) as compared with Ca^2+^ (Y857-L867) (Fig. 3d). The possibility is that ion interactions with the ion transporting TM3 helix rigidifies the nearby TM4 helix. An alternative possibility is that D157, E159, E862, and E866 residues (located on the 3L4 and 9L10 loops) ligate either Ca^2+^ or Na^+^, where the identity of the bound ion dictates the regulatory response of mammalian NCX.

### MD simulations reveal a Ca^2+^-induced population shift of conformational states in full-size NCX1.4

Even though HDX-MS provides indispensable dynamic information on the apo and ion-bound states, these data are at the primary sequence level; thus, they lack the spatial resolution to characterize the associated conformational transitions even when a static X-ray or cryo-EM structure is available. Since the region connecting TM5 with CBD1, encompassing the THB module, was not resolved in the cryo-EM structures we sought to explore the conformational transitions associated with the allosteric interactions of Ca^2+^ with the regulatory CBD1 and CBD2 domains in full-size NCX1.4 using MD simulations. In brief, full-size apo NCX1.4 was modeled based on the cryo-EM structure of the “inactivated” NCX1.1^49^, and the transmembrane region was embedded in a POPC bilayer. For the Ca^2+^ bound form, ions were introduced to the binding sites of CBD1 (four sites) and CBD2 (two sites) based on their available crystal structures (PDB 2DPK and 2QVM)^23,24^.

**Fig. 5:**
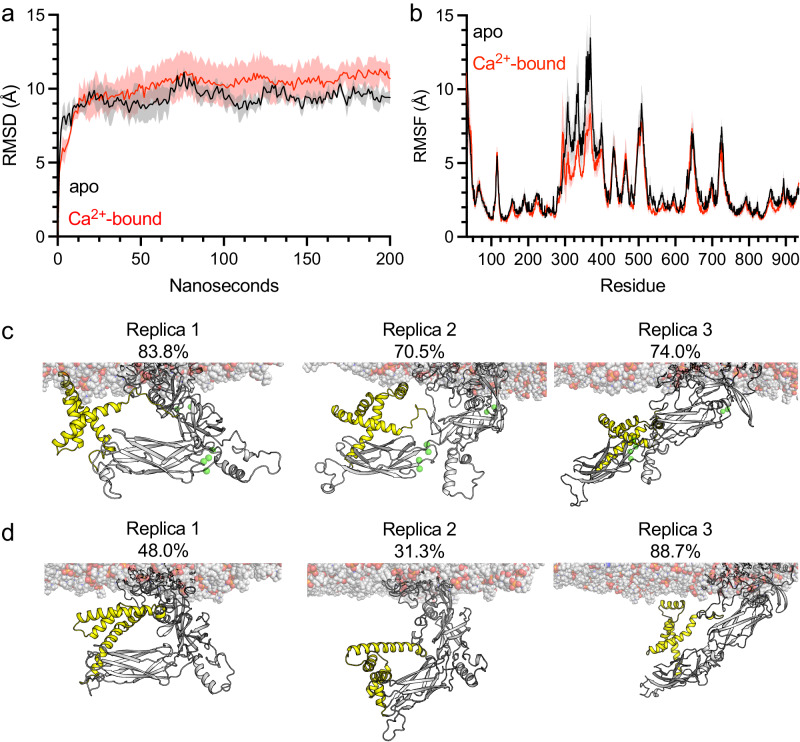
Molecular dynamics simulations of NCX1.4. **a** Average root mean square deviation (RMSD) of the apo (black) and Ca^2+^-bound (red) states along the simulation trajectory. **b** Average per residue root mean square fluctuation (RMSF). Shaded areas denote the SD. (**c**, **d**) Representative structures of the most abundant cluster in each replica, with its frequency along the trajectory of the Ca^2+^-bound (**c**) or apo (**d**) states. Lipids are denoted as spheres, whereas the protein is shown in cartoon representation. Residues 300–400 are shown in yellow.

All the simulations performed here have reached convergence, as reflected by the plateau in root mean square deviation values (Fig. 5a, Supplementary Table 1)^60^. Intriguingly, the per-residue root mean square fluctuation values, representing their spatial fluctuation relative to their average position along the simulation, diverge between the apo and Ca^2+^-bound states between residues 300–400, connecting TM5 with CBD1, and encompassing the THB (Fig. 5b)^20^. Clustering analysis revealed lower conformational heterogeneity in the Ca^2+^-bound state, where the most abundant cluster accounted for 76 ± 7% of the trajectory compared with 56 ± 29% in the apo state. In two out of three replicates of the Ca^2+^-bound state, the predominant conformation reveals a direct interaction between the predicted helix formed by residues 359–370, within the linker between TM5 and CBD1, and the membrane (Fig. 5c). Conversely, such a conformation is most abundant only in one simulation of the apo-state (Fig. 5d). Thus, the MD simulations support the notion that the Ca^2+^ binding to the two-domain CBD12 tandem in full-size NCX1.4 stabilizes the 5L6 loop and results in a population shift of conformational state assemblies, where conformations with tighter membrane interactions are more populated at dynamic equilibrium. A more dedicated experimental design is required to test the proposed working hypothesis.

## Discussion

Over the past decades, the NCX proteins have been extensively studied using biochemical, biophysical, electrophysiological, and structural biology approaches^8,9,35,47–50^. Although these studies have advanced our understanding of ion transport mechanisms in this physiologically crucial family of transporters, fundamental questions relating to their allosteric regulation remained unresolved. Indeed, even though it is widely accepted that ligand-induced long-range allosteric interactions are deep-rooted in the structure-dynamic features of protein molecules^53–55,61,62^ (like NCX), the underlying molecular mechanisms of allosteric regulation remain poorly understood. Therefore, the goal of the present study was to dissect the allosteric effects of Na^+^ and Ca^2+^ in the context of active full-length mammalian NCX.

To investigate the allosteric effects of Ca^2+^ or Na^+^, the apo, Ca^2+^-, and Na^+^-bound forms of purified NCX1.4 (Fig. 1c, d) were analyzed using HDX-MS (Figs. 2–4). Importantly, the HDX profile of apo NCX1.4 is consistent with the structural model (based on the cryo-EM structure of NCX1.1); it suggests that the protein maintains its activity and structural integrity following detergent solubilization. In addition, we obtained high sequence coverage (77%, excluding the SPS) for NCX1.4, including at the transmembrane segments encompassing the ion transport sites (Supplementary Figs. 1 and 2). In general, the experimental platform, developed here, can be applied for the HDX-MS analysis of ligand-induced effects on the backbone dynamics in mammalian NCX variants exhibiting different regulatory profiles. This seems to be an especially encouraging approach since NCX1, NCX2, and NCX3 isoform/splice variants exhibit ~70%^8,22,34^.

Since the cryo-EM structures were obtained only in the presence of Ca^2+^-bound CBD1, either in the inactive or active state^49,50^, the present HDX-MS analysis of full-length NCX1.4 sheds light on the structural role of Ca^2+^ binding to the high-affinity Ca^2+^ sensor at CBD1. Our results align well with previous biophysical investigations of the two-domain (CBD12) constructs (obtained from different NCX isoform/splice variants), revealing that high affinity Ca^2+^ binding to CBD1 (at the interface with CBD2) rigidifies the backbone dynamics of both CBDs and interdomain linker that affects the frequency rather the amplitude of interdomain CBD movements^39,43–45^. Thus, the currently available information strongly supports the notion that the Ca^2+^-mediated tethering of the CBDs restricts interdomain movements of CBDs in full-size NCX1.4 to “transmit” the allosteric message to membrane-associated TM segments. We suggest that the Ca^2+^-mediated rigidification of the CBDs movements represents a common mechanism for mammalian NCX regulation^35^, where the dynamic features of interdomain movements can be “secondarily” modulated by varying the exons compositions in isoform/splice variants^35,39,43–45^.

Notably, previous studies have shown that the Ca^2+^-driven structural tethering of CBDs results in a 50-fold deceleration in the Ca^2+^ off-rates from a high-affinity Ca^2+^ sensor^36^, where the dissociation rates of trapped (occluded) Ca^2+^ from the two-domain interface dramatically varies among NCX isoform/splice variants^37,40^. The Ca^2+^ occlusion at the two-domain interface plays a primary physiological role, since the Ca^2+^ off-rates of occluded Ca^2+^ correlate with slow inactivation kinetics monitored upon the cytosolic Ca^2+^ removal^46,47^. Consistent with previous studies showing that the exon composition of the splicing segment at CBD2 modulates the Ca^2+^ dissociation rates from CBD12^37,40^, the present HDX-MS analysis reveals Ca^2+^-dependent rigidification of the N-terminus of CH1 on the FG-loop of CBD2 (which is part of the splicing segment), residing in close vicinity with the high-affinity Ca^2+^ binding sites of CBD1 (Fig. 3a, b). Our findings also corroborate the CH1 conformation observed in the cryo-EM structures of NCX1.1^49^ and NCX1.3^50^, suggesting that this region is indeed stably packed against CBD1 under native conditions and that the observed conformational state is not solely imposed by the Fab antibody or inhibitor molecule used for structure determination.

Conceptually, a fractional shift in the existing conformational assemblies can couple the ligand-induced allosteric interactions between the regulatory and functional domains in different types of proteins^61–63^. Extensive biophysical studies of isolated CBD12 strongly supported the population shift mechanism^45^, further reinforced by present HDX-MS analysis and the MD simulations of full-length NCX1.4 and provided additional insights into the underlying structure-dynamic mechanism of ion-induced allosteric regulation. In this respect, the previous structural, functional, and mutational studies revealed that the dynamic features of interdomain CBDs’ movements are controlled by the interdomain linker (that links the CBDs) and the exon composition within the splicing segment at CBD2^37,44,45,64^. Although these research avenues have elucidated how the Ca^2+^-driven signal becomes primarily decoded, modified, and coupled at CBDs, the structure-dynamic mechanisms underlying the long-range allosteric interactions in the full-size mammalian NCX remained elusive.

Although the HDX-MS analysis of full-size NCX1.4 presented here shows the Ca^2+^-induced rigidification of both CBDs, we could not detect any backbone rigidification path that spends for allosteric signal propagation from the CBDs to transmembrane segments (Fig. 3b). These data further support the dynamic nature of allosteric regulation, which couples the interdomain CBD movements and Ca^2+^ occlusion at the two-domain interface^36,45^. Strikingly, the MD simulations presented here propose a novel role for the TM5-CBD1 linker region, largely owing to a α-helix structure (Fig. 5). According to this model, the side chain moieties of the ^359^-AFYRIQATRLMT-^370^ sequence (forming an amphipathic helix) may favorably anchor the 5L6 to the membrane upon Ca^2+^ binding to the CBDs. It is possible that the present HDX-MS analysis cannot detect these side-chain interactions with the membrane, since the HDX-MS techniques can monitor the backbone dynamics, not the side-chain interactions.

**Fig. 6:**
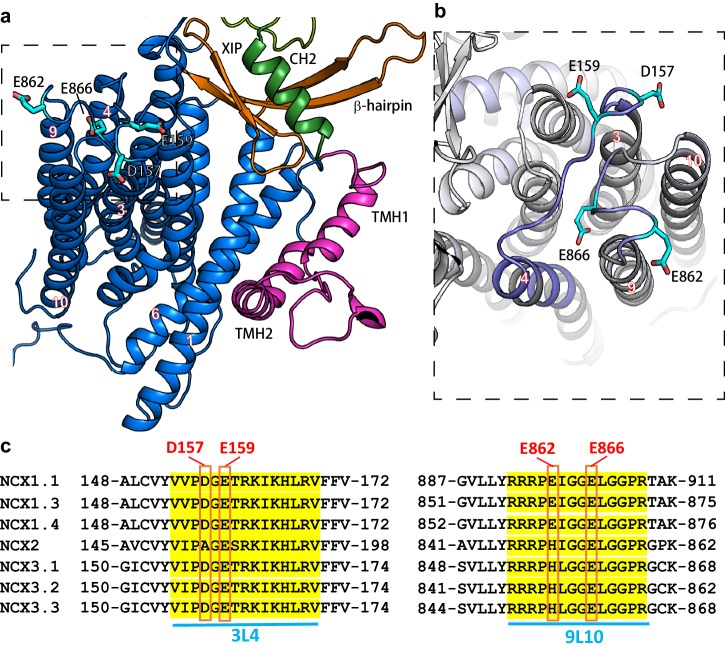
The putative regulatory site at the intracellular surface of NCX1.4. **a** The transmembrane domain and the inactivation module in cartoon representation. The location of the CH2 (in green), β-hub (in orange), TMH1 and TMH2 (in magenta), and transmembrane (TM) (in blue) domains are near the putative regulatory sites (for Na^+^ and Ca^2+^ binding), located on the cytosolic 3L4 and 9L10 loops. Potential ion-binding residues are shown as sticks and colored cyan. **b** A zoom perspective of the proposed ion-binding site. The displayed region is framed by a rectangle in (**a**) and has been reoriented for clarity. The residues are colored according to the differential HDX signal as in Fig. 3d, and the potential cation binding residues are shown as sticks and colored cyan. **c** Sequence alignment of the 3L4 and 9L10 loops are presented for the indicated NCX variants. The NCX1, NCX2, and NCX3 variants include four, two, and three negatively charged residues, respectively, thereby suggesting that the ion-ligation variations at respective sites may account for the distinct regulatory effects induced by Na^+^ in NCX variants.

Besides the Ca^2+^-dependent rigidification of CBD1 and CBD2, an interesting observation is that Ca^2+^ stabilizes the N-terminal portion of CH2 (Fig. 3). This observation is hard to reconcile with a previous proposal, suggesting that Ca^2+^ binding to CBD2 destabilizes nearby structures at the interface of the regulatory cytosolic domains and ion-transporting transmembrane helices^49^. Intriguingly, our results expose two regions exhibiting a similar response to Ca^2+^ (Fig. 3) or Na^+^ (Fig. 4) at the cytosolic side of the transmembrane domain, thereby suggesting the possible involvement of relevant sites in shaping the ion-induced allosteric signals. These regions include the TMH2-TM1-TM6 cluster (Figs. 3c and 4c) and the TM3-3L4-TM4/TM9-9L10-TM10 cluster (Figs. 3d and 4d). The HDX signals observed at these regions represent a primary interest concerning allosteric regulation since they are positioned at the interface between the cytosolic β-hub (including the auto-inhibitory XIP domain) and the membrane helical domains managing the ion-transport events (Fig. 6).

Even though our HDX-MS findings may represent a rebound (indirect) effect of Na^+^ (or Ca^2+^) interactions with the ion-transporting TM3 helix and the subsequent stabilization of the nearby TM4 helix, some alternative possibilities should be seriously considered for Na^+^-induced inactivation. In general, the Na^+^-induced inactivation of NCX1 and NCX3 refers to a time-dependent decay of NCX-mediated ion-exchange currents due to increased levels of cytosolic Na^+^^28^. Originally, it was proposed that the Na^+^-induced inactivation originates from the occupancy of the transport site(s) by cytosolic Na^+^, while driving the protein into an “inactivated state”^28^. Even though the cryo-EM structure of NCX1.1 inactivated state (8SGJ) refers to the occupation of three binding sites (S_ext_, S_int_, and S_Ca_) by Na^+^, only a weak density was observed at S_Ca_, and no signal was detected at the other binding sites^49^. Therefore, the occupation of three binding sites (S_ext_, S_int_, and S_Ca_) by 3Na^+^ ions and of S_mid_ by a water molecule in the IF state of NCX1.1^49^ is modeled based on the well-defined ion-ligation geometry, revealed by high-resolution crystal structures of NCX_Mj in the OF state^10,11^.

Even though the NCX1, NCX2, and NCX3 isoforms all contain identical ion-coordinating residues for Na^+^ or Ca^2+^ binding/transport, some structural variances at XIP and CBD2 (in conjunction with other structural elements) may contribute to the lack of Na^+^-induced inhibition in NCX2 (unlike to NCX1 and NCX3). Recently determined cryo-EM structures of mammalian NCX1.1^49^ and NCX1.3^50^ provide new opportunities for structure-based functional analyses of mutants toward resolving the underlying mechanism of Na^+^-induced inactivation mode. Interestingly, early mutational studies^19,31,65^ already have suggested that Na^+^-induced inactivation may involve multiple topological entities that are located outside the ion-transporting domains although the identification of Na^+^ binding inactivation site mediating the allosteric regulation of mammalian NCXs remained unresolved^4–9^. Based on the present HDX-MS data (Figs. 3c, d and 4c, d), a new mechanism for Na^+^-induced allosteric inhibition is proposed here, where two different regions in NCX1.4 may bind Na^+^ to mediate the Na^+^-induced inactivation.

The first region that could be involved in the Na^+^-induced allosteric regulation of NCX1.4 is the TMH2-TM1-TM6 cluster (Figs. 3b, c and 4b, c). Strikingly, TMH2 (the palmitoylation helix) interacts with the sliding bundle (TM1/TM6) in the close vicinity of the ion-binding TM2/TM3 helices. The mechanistic significance of the present HDX-MS analysis is that the ion-dependent rigidification of TMH2-TM1-TM6 can affect the rate-limiting transitions associated with alternating access. Interestingly, the NCX_Mj (and other prokaryotic NCXs) lack the TMH2 domain, thereby suggesting that in mammalian NCXs the dual interaction of TMH2 with the TM1/TM6 bundle (from one side) and the ion-transporting TM2/TM3 entity (on the other side) may account (at least partially) for huge differences in the transport rates between the mammalian and prokaryotic NCXs^12,57–59^. Another important point is that the palmitoyl moiety, attached to the C703 of NCX1.4 (C735 with the N-terminal 32 residues representing the signal peptide sequence)^25,49^ can predefine the positioning of the TMH2-TM1-TM6 cluster within the membrane, while the dual interactions of TMH2 with TM1/TM6 and TM2/TM3, can modulate the ion transport rates. Thus, Na^+^ or Ca^2+^ interactions with TMH2-TM1-TM6 can govern (at least partially) the ion-dependent allosteric regulation of mammalian NCXs.

The second region that could be involved in the Na^+^-induced regulation refers to the TM3-3L4-TM4 (Y152-A179) and TM9-9L10-TM10 (Y857-L876) segments since Na^+^ or Ca^2+^ rigidify this region (Figs. 3 and 4). Notably, this region is near the ion-transporting helices TM7 and TM8, suggesting that allosteric interactions may affect ion transport rates in mammalian NCXs. We posit that Na^+^ and Ca^2+^ binding to the 3L4 (D157 and E159) and 9L10 (E862 and E866) segments can form dual interactions with the ion-transporting TM7/TM8 cluster and the XIP-containing β-hub (on the other side) (Fig. 6). Despite the shared reduction in deuterium uptake, the actual conformation of this region may differ in the Na^+^ or Ca^2+^ bound states, contributing to the diverse regulatory response. Thus, the present HDX findings support the possibility that the 3L4-9L10 loop cluster (containing D157, E159, E862, and E866 residues in NCX1), may provide a structural basis for Na^+^-induced inactivation. Even though there are some differences in the XIP/CBD2/β-hub sequences among NCX1-3 isoforms, it is essential to note that NCX1, NCX2, and NCX3 isoforms contain four, two, and three carboxyl residues (respectively) at putative Na^+^ binding site on the 3L4 and 9L10 loops (Fig. 6c). These “subtle” structural variances may shape the affinity and kinetics of Na^+^-induced inactivation among three NCX isoforms. Due to these structural variances at the 3L4-9L10 loop cluster, NCX1 can exhibit a “higher affinity” for Na^+^-induced inactivation (IC_50_ ≈ 5 mM) than NCX3 (IC_50_ = 20-40 mM), whereas NCX2 cannot bind Na^+^ at the putative site (and thus, cannot mediate Na^+^-induced inactivation).

The newly proposed mode of Na^+^-induced inactivation is remarkable in context with previous findings revealing that H165 (located on the 3L4 loop next to D157 and E159) plays a key role in proton-dependent inactivation of NCX1.1^66,67^. Unfortunately, the 3L4 and 9L10 loops were not well resolved in the cryo-EM structures. Moreover, previous studies have shown that the mutation of D101 and H165 (located at the cytoplasmic ends of TM2 and TM4) eliminates Na^+^-induced inactivation in NCX1.1^63,64^, or the mutation of three residues located close to the extracellular end of TM2 (H124, F126, and E130) affect the kinetics of Na^+^ inactivation^65,68^. Notably, the inhibitory XIP domain somehow relates with the Na^+^-induced inactivation mechanism since single point mutations within the XIP segment can either abolish or enhance Na^+^-induced inactivation^19,31,47^. Even though the cryo-EM structures^49,50^ did not detect any Na^+^-binding sites, the present HDX-MS analysis suggests that two different regions in NCX1.4 may bind regulatory Na^+^ or Ca^2+^ thereby balancing a dynamic equilibrium (at steady-state) between the active and inactive states. This working hypothesis can be analyzed by structure-based testing of mutational effects on the Na^+^-induced inactivation using the previously established patch-clamp protocols^7,23,24,46,47,65,67^.

Another structural element that could be tightly coupled to the Na^+^-induced regulation, is a short palmitoylation helix, TMH2. Since the palmitoylation of NCX1.1 affects the extent of Na^+^-induced inactivation^69^, the Na^+^ or Ca^2+^ promoted rigidification of the TMH2-TM1-TM6 cluster, found here (Figs. 3 and 4) might have a mechanistic and physiological significance. Even though it remains unclear whether the HDX-MS findings reflect the direct interactions of Na^+^ with the TMH2-TM1-TM6 cluster or indirect interactions (associated with Na^+^ binding to transport sites), the TMH2-TM1-TM6 entity may serve as a Na^+^ binding site that can mediate Na^+^-induced inactivation. A more dedicated patch-clamp analysis of NCX mutants is required to examine the interaction of Na^+^ with the putative regulatory site(s).

Surprisingly enough, the “inactivation module” (as assigned by the cryo-EM study of NCX1.1^49^) showed relatively high deuterium uptake (Fig. 2), which was unaffected by either Ca^2+^ (Fig. 3) or Na^+^ (Fig. 4). However, the CH2-β-hub interactions remained stable throughout our MD simulations, either in the presence or absence of Ca^2+^ bound to the CBD12 sites (Fig. 6). Taken together with the cryo-EM structures, the activation/inactivation modules (controlled by Ca^2+^ and Na^+^ binding to respective regulatory sites) may merely shift the dynamic equilibrium between the active and inactive states. It is essential to note the cryo-EM structures of NCX1.1 were obtained with a bound Fab antibody (in the vicinity of CBD1), since without the Fab antibody, the 5L6 mobility was increased, thereby precluding the detection of a stable structure by cryo-EM^49^. Thus, the Fab antibody probably stabilizes a specific conformation (most probably, in the inactive state), although the physiological relevance of Fab-bound conformations is currently difficult to evaluate.

In summary, the present findings provide valuable information on the Ca^2+^- and Na^+^-induced conformational changes associated with the allosteric regulation of full-size mammalian NCX1.4, thereby revealing a structure-dynamic basis for the ligand-induced population shift of conformational assemblies. In combination with structure-based functional analysis of relevant mutants using patch-clamp methodologies, the currently developed approaches provide a basis for identifying putative ion binding site(s) and for segregating specific conformational transitions associated with distinct regulatory ligands (PIP_2_, negatively charged lipids, cholesterol, and post-translational palmitoylation). These developments can further promote structure-based screening, design, and development of “drug-like” ligands. Moreover, the newly developed experimental procedures and computational protocols confidently establish a good platform for detecting and characterizing the ligand-induced conformational changes in the NCX1, NCX2, and NCX3 isoform/splice variants, exhibiting ~70% sequence identity. The follow-up investigations may allow a structure-based pharmacological targeting of desired NCX isoform/splice variants (expressed in a tissue-specific manner) involved in disease related conditions, which in turn can provide new opportunities for clinically related drug development.

## Methods

### DNA cloning

The *SLC8A1* sequence of *Canis lupus familiaris* (Uniprot ID: P23685) was used for the DNA cloning of the NCX1.4 construct. The DNA sequence of NCX1.4 (including the A and D exons) was inserted into the pcDNA3.4 expression vector. A 10xHis-tag was attached to the C-terminus of the NCX1.4 sequence. Codon optimization was performed to avoid negative cis-acting sites (such as splice sites and TATA-boxes, etc), and the GC content was adjusted to prolong the mRNA half-life time of encoded NCX1.4 and to increase the mRNA access to ribosomes. The codon adaptation index of 0.94 was achieved in designing the codon optimization, which is desirable for sustaining elevated levels of NCX1.4 protein expression in the HEK293F cells. A construct bearing a C-terminal GFP-fusion (NCX1.4/GFP/10xHis tag) was used to evaluate the DNA/PEI transfection efficiency in the HEK293F cell suspension - this construct was not used ether for NCX1.4 purification or its HDX-MS analysis.

### NCX1.4 overexpression in HEK293F cells

Suspension-adapted HEK293F cells were grown in either SMM-293TII (Sino Biological) or Freestyle 293 (cat. No. 12338018, Invitrogen) expression medium, supplemented with 2 mM L-glutamine (Sigma). Next, 25 ml of cell culture suspensions were placed in 125 ml flasks and then continuously shaken at 125 rpm in an incubator (37 ˚C and 5% CO_2_) using an orbital shaker (LG-TAB002125, Lifegene, Switzerland). To overexpress the milligram quantities of NCX1.4 in HEK293F, a transient transfection protocol relying on 40 kDa linear polyethyleneimine (PEI, Polysciences) was explored. One day before transfection, high-density cells (0.9–1.2 × 10^6 ^ml^–1^) were diluted into fresh medium (4 × 280 ml). Transfection-grade plasmid DNA was mixed with PEI (at a ratio of 1:3) and vortexed at room temperature for 15–30 min. Transfection was initiated by diluting the DNA/PEI mixture into the cell suspension while reaching a final concentration of 0.5 µg DNA/ml. After 24 h of transfection, a pre-heated SMM 293-TII or Freestyle medium was added to the suspension, and cells were allowed to grow for an additional 24 h. Typically, 30–50% of transfection efficiency was achieved by using the DNA/PEI protocol. Finally, cells were harvested by centrifugation at 10,000 × *g* for 10 min and cell pellets were either directly used for cell disintegration and isolation of cell membranes or cells were flash-frozen in liquid nitrogen and stored at –80 ˚C until cell disruption and cell membrane isolation (see below). A typical yield for cell growth of HEK293F cells was ~5 g of cells per 1 L of medium.

### NCX1.4 purification

The HEK293F cells (20–35 g), obtained from 4 to 6 L of cell suspension, were diluted with a buffer containing 20 mM Tris pH 7.5, 150 mM NaCl, 2 mM MgCl_2_, 1 mM EGTA, and 1 mM DTT, supplemented with DNase (10 µg/ml) and protease inhibitor cocktail, Xpert (GenDEPOT), 1 mM PMSF Sigma, USA, and 1 mM benzamidine (Sigma USA). The cell suspension was homogenized and gently stirred for 15–20 min at 4 °C. The HEK293F cells were disrupted three times after passing the cell suspension through the EmulsiFlex-C3 device (Avestin, Inc.) at 15,000–20,000 psi. Isolated cell membranes were collected by centrifugation at 60,000 × *g* for 1 h (4 °C) and then washed by centrifugation in the same buffer. The membrane pellet was solubilized with DDM-containing buffer (20 mM Tris pH 7.5, 300 mM NaCl, 10%, glycerol, 20 mM DDM, and 2 mM CHS) containing protease inhibitors (DDM and CHS were from Anatrace). After 2 h of incubation at 4 °C, insoluble materials were removed by centrifugation at 60,000 × *g* for 30 min. The clear supernatant was mixed with 1–2 ml TALON resin (Takara, Japan) at 4 °C, and after 12–18 h, the slurry was transferred to the gravitation column, after which the effluent was discarded. The column was washed with 50–100 ml of 20 mM Tris pH 7.5, 300 mM NaCl, 10% glycerol, 1 mM DDM, 0.1 mM CHS, and 10–20 mM imidazole. Before the NCX1.4 elution, the column was flushed with 20 ml Choline-Cl buffer (20 mM Tris pH 7.5, 50 mM Choline-Cl, 4 mM DDM, and 10–20 mM imidazole). Finally, NCX1.4 was eluted with 300 mM imidazole containing Choline-Cl buffer (20 mM Tris pH 7.5, 50 mM Choline-Cl, and 1 mM DDM). Fractions containing NCX1.4 were collected, concentrated (Amicon, 100 kD cut-off filter), and then subjected to SEC chromatography using a Superdex SD200 Increase column, 10/300 GL (Cytiva) equilibrated with 20 mM Tris pH 7.5, 50 mM Choline-Cl, 0.5 mM EDTA, and 1 mM DDM. To confirm the NCX1.4 identity, the purified samples were analyzed using SDS-PAGE and Western Blots (with His-tag antibodies). The eluted fractions containing NCX1.4 were collected, concentrated to 1–2 mg protein/ml, flash-frozen in liquid nitrogen, and stored at −80 °C until use.

### Proteoliposomes reconstitution

For preparing the NCX1.4-reconstituted proteoliposomes, 50–100 µl of purified NCX1.4 protein (~0.5 mg/ml) was added to a lipid mix of POPE:POPG (Avanti Polar Lipids) at a ratio of 3:1 (w/w), while maintaining a protein-to-lipid ratio of 1:50–100 (w/w). After 30 min of incubation, detergent was removed by adding SM2 beads and with gentle agitation at 4 °C for 14–18 h. Finally, the reconstituted proteoliposomes were collected by the centrifugation of 1.5 ml Eppendorf tubes at 60,000 × *g* for 1 h, and pellets were suspended in a minimal volume of storage buffer (20 mM Tris-HCl, pH 7.5, and 100 mM CsCl). Flash-frozen aliquots of reconstituted proteoliposomes were stored at −80 °C until use. Before the assay of ion-flux activities, the proteoliposomes were briefly sonicated in a water-bath sonicator and loaded with 80–160 mM NaCl (or CsCl), and 0.5 mM EGTA at 35 °C for 2 h.

### NCX1.4-mediated ion-flux assays

The Na^+^/Ca^2+^ exchange reaction was assayed at 35 °C by measuring ^45^Ca^2+^ uptake in HEK293F cells or liposome-reconstituted vesicles containing the purified preparations of NCX1.4. The ^45^Ca^2+^-uptake was initiated by diluting Na^+^-loaded cells (20–30 mM NaCl) or proteoliposomes (80–160 mM) into the assay medium with 50–100 µM ^45^CaCl_2_ according to the established protocols^12–16^. The ^45^Ca^2+^-uptake (2–300 s) was quenched at desired time points by rapidly injecting a cold quenching buffer (with 5 mM EGTA) into the assay medium, and the intracellular (or intravesicular) ^45^Ca^2+^ content was determined by rapid filtration of the quenched samples through the GF/C filters (Tamar Ltd., Israel). The cells/proteoliposomes with trapped ^45^Ca^2+^ on the filter were washed several times with a cold quenching buffer, and finally, the filters were dried to perform scintillation counting. Cells or liposomes lacking NCX1.4 were used for blank assays to evaluate non-specific Ca^2+^ binding/transport. The non-specific (blank) signals were subtracted from the Ca^2+^-uptake signals in HEK293F cells (expressing NCX1.4) or in proteoliposomes (with a reconstituted NCX1.4). Blanks were obtained by adding 160 mM NaCl to the assay medium to prevent Na^+^/Ca^2+^ exchange and background (nonspecific) ^45^Ca^2+^ signals (bound to the filer) were subtracted from the ^45^Ca^2+^-uptake data.

### HDX-MS analysis of NCX1.4

The HDX-MS analysis of NCX1.4 was carried out in apo, Na^+^-, and Ca^2+^-bound states using experimental procedures we explored in previous studies^14,15^. The basic buffer contained 20 mM Tris-HCl pH 7.5, 0.5 mM EGTA, 0.25 mM DDM and 50–150 mM Choline-Cl to keep ionic strength constant in apo (150 mM), Na^+^ (100 mM + 50 mM Choline-Cl) and Ca^2+^ (5 mM + 150 mM Choline-Cl) samples. Deuterated buffers of identical composition with pD 7.5 (pH 7.1)^70^ were used for a 10-fold dilution of the protein stock solution (9.6 µM). The HDX-MS experiment was performed using a PAL HDR autosampler (CTC Analytics, Zwingen, Switzerland) operated by Chronos software (AxelSemrau, Sprockhoevel, Germany). Each HDX time point was prepared by mixing 5 µl of protein with 45 µl of the deuterated buffer. Five different time points, namely, the 20 s, 1 min, 5 min, 20 min, and 2 h were followed, and 20 s and 5 min were done in triplicate. The HDX reaction was quenched by an ice-cold 20 mM H_3_PO_4_ and 0.25 mM DDM in a ratio of 1:1. The exchange was conducted with two different batches, one at 4 °C and the other at 21 °C. Fully deuterated control was prepared to correct for deuterium loss during the analysis. Each sample was processed as follows: The acidified protein solution was injected onto a protease column containing co-immobilized pepsin and Nepenthesin-2^71^. Peptides were trapped on a trap column (SecurityGuard™ ULTRA Cartridge UHPLC Fully Porous Polar C18, 2.1 mm ID, Phenomenex, Torrance, CA), where they were desalted. Digestion and desalting were driven by 0.4% formic acid in water delivered by an Agilent 1260 Infinity II Quaternary pump (Agilent Technologies, Waldbronn, Germany) at 200 µL.min^-1^. Next, peptides were passed through an analytical column (Luna Omega Polar C18, 1.6 µm, 100 Å, 1.0 × 100 mm, Phenomenex, Torrance, CA) where they were separated using an acetonitrile gradient (10%–45%; solvent A: 0.1% FA in water, solvent B: 0.1%FA, 2% water in ACN). Separation was driven by the Agilent 1290 Infinity II LC system pumping at 40 µL.min^−1^. The outlet of the analytical column was directly interfaced to an ESI source of tims TOF Pro (Bruker Daltonics, Bremen, Germany) operating in MS1 mode with 1 Hz data acquisition. The entire LCMS setup was placed in a cooled compartment to prevent deuterium loss.

The LCMS data were peak-picked in DataAnalysis 5.3 and exported. Further processing was done in DeutEx^72^. The HDX-MS data was visualized using MSTools^73^ and PyMol. The peptides, raised from pepsin/nepenthesin-2 digestion, were identified using an LCMS system identical to what was used for HDX. However, the mass spectrometer operated in data-dependent MSMS mode with PASEF enabled. The N-terminus amino acid of each peptide was ignored or calculated from the difference between the two overlapping peptides. Tandem mass spectra were searched using MASCOT (v. 2.7, Matrix Science, London, UK) against a database containing sequences of NCX1.4, acid proteases, and cRAP.fasta (https://www.thegpm.org/crap/). The search parameters were as follows: precursor tolerance 10 ppm, fragment ion tolerance 0.05 Da, decoy search enabled with FDR <1%, IonScore > 20, and peptide length >5. The mass spectrometry data have been deposited to the ProteomeXchange Consortium via the PRIDE^71,74^ partner repository with the dataset identifier PXD047098.

### Structural modeling

Our structure is based on PDB ID 8SGJ (the inactive form of the human NCX1.1). To model the missing loop between TM5 and CBD1 (residues 281–401 in NCX1.4), full-length NCX1.4 was modeled using AlphaFold^75^ and aligned to PDB 8SGJ^49^. Then, the modeled missing region was copied using PyMol (Schrödinger LLC) to 8SGJ. Small missing regions from the N- and C-termini were similarly added. This structure was prepared by the protein preparation wizard of the Maestro suite (Schrödinger LLC, version 2020–3), including options for filling loops and filling residues, at pH 7.0 ± 1.0. This protocol adds missing hydrogen atoms, optimizes the hydrogen-bonding network, and performs restrained minimization^76^. Then, this structure was used as a template to model *Canis familiaris* NCX1.4 using Prime (Schrödinger LLC) in the inactive state. The sequence alignment was edited according to the Clustal Omega webserver^77^. All the parameters remained at the default values. For MD simulation analysis of Ca^2+^-bound NCX1.4, all six Ca^2+^ binding sites were occupied by Ca^2+^ at CBD1 (Ca1-Ca4) and CBD2 (CaI and CaII), as available from the Ca^2+^-bound structures of CBD1 and CBD2 (PDB 2DPK and 2QVM, respectively)^23,24^.

### MD simulations

All simulations were performed using the Maestro suite (Schrödinger LLC, version 2020–3). To perform MD simulations, the ‘inactive’ (apo) and ‘active’ (Ca^2+^-bound) structural models of NCX1.4 (see above) were prepared using the protein preparation wizard at pH 7.0 ± 1.0. The system setup tool was used to embed the membrane-spanning regions (residues 37–71, 99–123, 130–154, 166–188, 197–219, 731–751, 755–775, 805–825, 837–857, and 875–895, defined using the OPM database)^78^ in a POPC lipid bilayer. The systems were solvated using the TIP3P solvent model^79^. K^+^ or Cl^-^ ions were added to neutralize the charge and to obtain a final salt concentration of 150 mM. All MD simulations were performed using Desmond with the OPLS3e force field^80^. The simulations were conducted under a Langevin temperature and pressure control, using periodic boundary conditions with particle-mesh Ewald electrostatics with a 12 Å cutoff for long-range interactions. The systems were equilibrated using the default relaxation protocol and finally, the production simulations were conducted in triplicates (each starting from a random seed) for 200 ns with a constant pressure of 1 atm and a constant temperature of 300 K. The results were manually inspected and analyzed using the Maestro suite. Clustering analysis was performed with a 3 Å cutoff.

### Statistics and reproducibility

Two-group comparisons were performed using an unpaired *t* test assuming Gaussian distribution with the GraphPad Prism version 9 program (GraphPad Software). No statistical methods were used to predetermine sample sizes. Required experimental sample sizes were chosen according to common practice in protein biochemistry (at least three independent experiments). Statistical analysis was limited to determining mean ± SEM.

### Reporting summary

Further information on research design is available in the Nature Portfolio Reporting Summary linked to this article.

### Supplementary information

Supplementary Material

Description of Additional Supplementary Files

Supplementary Data 1

Supplementary Data 2

Reporting Summary

## Data Availability

All data related to MS analyses are deposited in PRIDE - ProteomeXchange under identifier PXD047098. This includes the raw data as well as readable numerical sources for data behind Figs. 2, 3, and 4 and Supplementary Figs. 1 and 2. Supplementary Data 1 includes the source data behind Fig. 1c, d, and Fig. 5. Supplementary Data 2 includes PDB files of the initial and final coordinates of the MD simulations. Any other relevant data are available from corresponding authors upon request.
