# Supplementary material for: Structural dynamics of Na+ and Ca2+ interactions with full-size mammalian NCX
Source: Commun Biol. 2024 Apr 16;7:463. doi: 10.1038/s42003-024-06159-9 (PMC11021524; doi:10.1038/s42003-024-06159-9)
Supplement: Supplementary file 1 — Supplementary Material [file 42003_2024_6159_MOESM1_ESM.pdf]

Supplementary information

Structural dynamics of Na^{+} and Ca^{2+} interactions with full-size mammalian NCX

Moshe Giladi,[1,3,*,#] Lukáš Fojtík,[2,4,*] Tali Strauss,[1,*] Benny Da'adoosh,[5] Reuben Hiller,[1] Petr Man[2,#], and Daniel Khananshvili[1,#]

[1]Department of Physiology and Pharmacology, Faculty of Medicine, Tel-Aviv University, Tel Aviv 69978, Israel

[2]Institute of Microbiology of the Czech Academy of Sciences, Division BioCeV, Prumyslova 595, 252 50 Vestec, Czech Republic

[3]Tel-Aviv Sourasky Medical Center, Tel Aviv 6423906, Israel

[4]Department of Biochemistry, Faculty of Science, Charles University, 128 00, Prague, Czech Republic

[5]Blavatnik Center for Drug Discovery, Tel Aviv University

[*]These authors contributed equally

[#]Correspondence: moshegil@post.tau.ac.il, pman@biomed.cas.cz, or dhanan@tauex.tau.ac.il

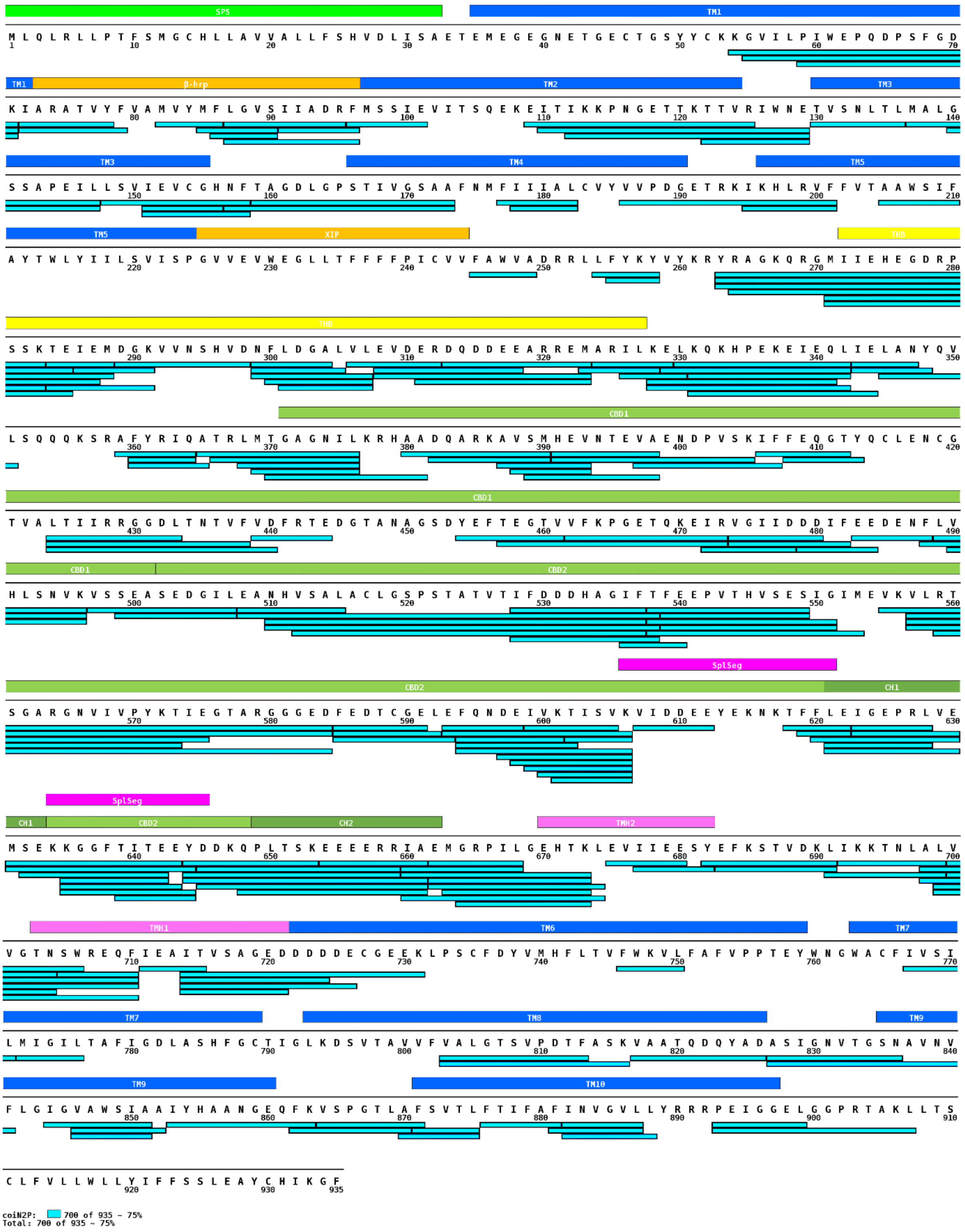

Supplementary Figure 1. Sequence coverage in HDX-MS experiments. The coverage map shows peptides generated after online digestion using immobilized pepsin/nepenthesin-2 under HDX-MS conditions.

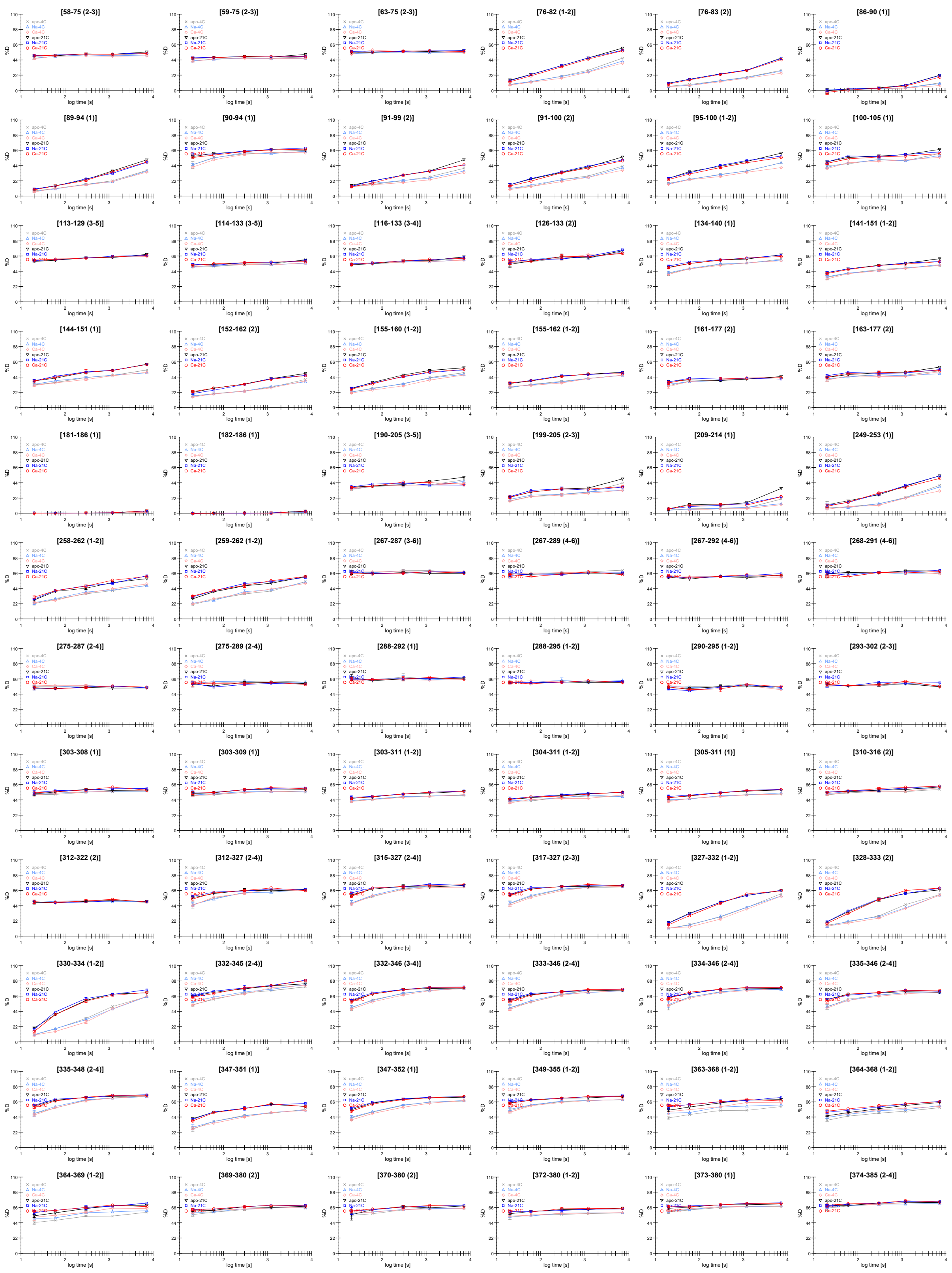
[58-75 (2-3)]
[59-75 (2-3)]
[63-75 (2-3)]
[76-82 (1-2)]
[76-83 (2)]
[86-90 (1)]
[89-94 (1)]
[90-94 (1)]
[91-99 (2)]
[91-100 (2)]
[95-100 (1-2)]
[100-105 (1)]
[113-129 (3-5)]
[114-133 (3-5)]
[116-133 (3-4)]
[126-133 (2)]
[134-140 (1)]
[141-151 (1-2)]
[144-151 (1)]
[152-162 (2)]
[155-160 (1-2)]
[155-162 (1-2)]
[161-177 (2)]
[163-177 (2)]
[181-186 (1)]
[182-186 (1)]
[190-205 (3-5)]
[199-205 (2-3)]
[209-214 (1)]
[249-253 (1)]
[258-262 (1-2)]
[259-262 (1-2)]
[267-287 (3-6)]
[267-289 (4-6)]
[267-292 (4-6)]
[268-291 (4-6)]
[275-287 (2-4)]
[275-289 (2-4)]
[288-292 (1)]
[288-295 (1-2)]
[290-295 (1-2)]
[293-302 (2-3)]
[303-308 (1)]
[303-309 (1)]
[303-311 (1-2)]
[304-311 (1-2)]
[305-311 (1)]
[310-316 (2)]
[312-322 (2)]
[312-327 (2-4)]
[315-327 (2-4)]
[317-327 (2-3)]
[327-332 (1-2)]
[328-333 (2)]
[330-334 (1-2)]
[332-345 (2-4)]
[332-346 (3-4)]
[333-346 (2-4)]
[334-346 (2-4)]
[335-346 (2-4)]
[335-348 (2-4)]
[347-351 (1)]
[347-352 (1)]
[349-355 (1-2)]
[363-368 (1-2)]
[364-368 (1-2)]
[364-369 (1-2)]
[369-380 (2)]
[370-380 (2)]
[372-380 (1-2)]
[373-380 (1)]
[374-385 (2-4)]
apo-4C
Na-4C
Ca-4C
apo-21C
Na-21C
Ca-21C
%D
log time [s]

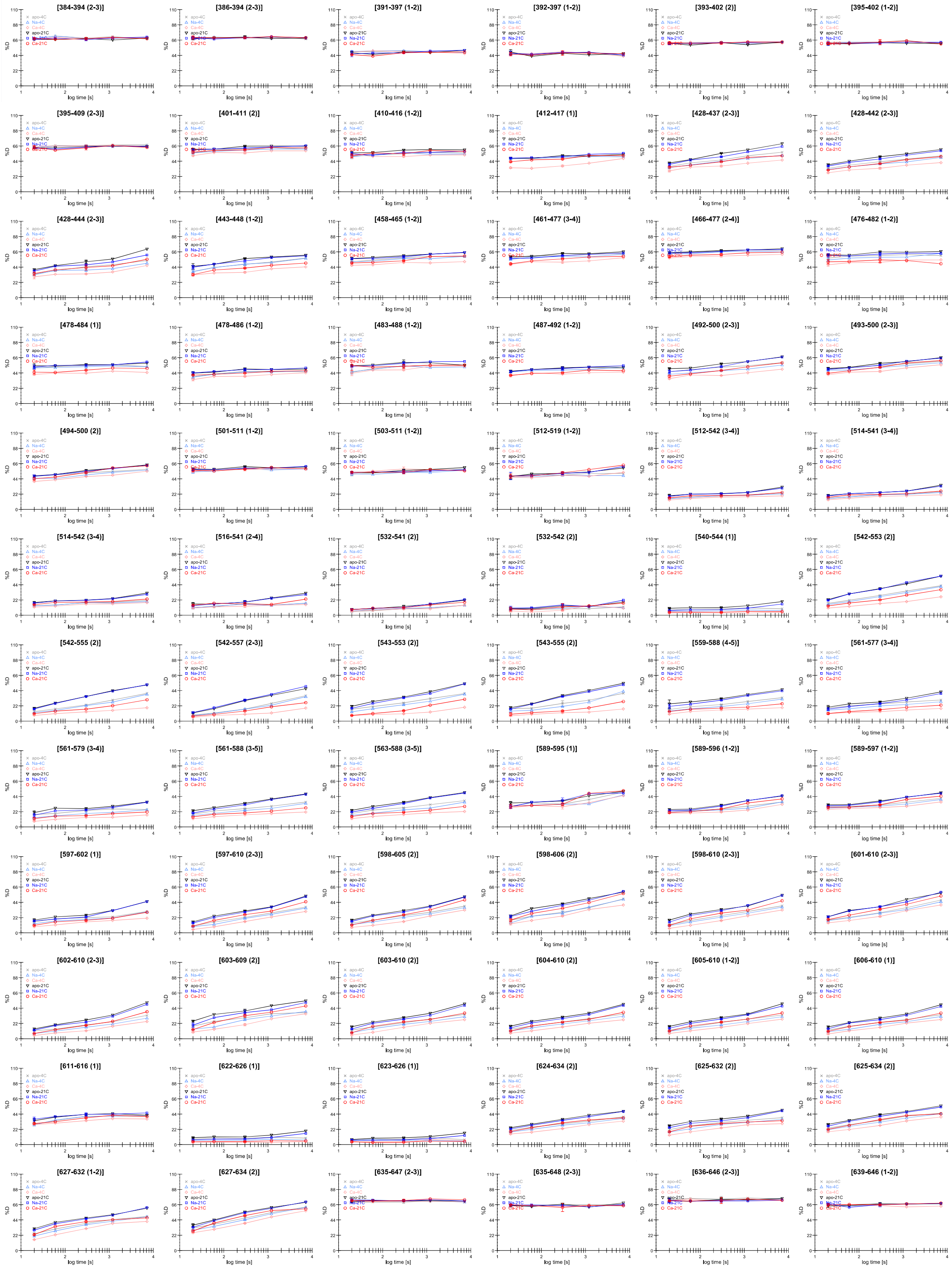
[384-394 (2-3)]
[386-394 (2-3)]
[391-397 (1-2)]
[392-397 (1-2)]
[393-402 (2)]
[395-402 (1-2)]
[395-409 (2-3)]
[401-411 (2)]
[410-416 (1-2)]
[412-417 (1)]
[428-437 (2-3)]
[428-442 (2-3)]
[428-444 (2-3)]
[443-448 (1-2)]
[458-465 (1-2)]
[461-477 (3-4)]
[466-477 (2-4)]
[476-482 (1-2)]
[478-484 (1)]
[478-486 (1-2)]
[483-488 (1-2)]
[487-492 (1-2)]
[492-500 (2-3)]
[493-500 (2-3)]
[494-500 (2)]
[501-511 (1-2)]
[503-511 (1-2)]
[512-519 (1-2)]
[512-542 (3-4)]
[514-541 (3-4)]
[514-542 (3-4)]
[516-541 (2-4)]
[532-541 (2)]
[532-542 (2)]
[540-544 (1)]
[542-553 (2)]
[542-555 (2)]
[542-557 (2-3)]
[543-553 (2)]
[543-555 (2)]
[559-588 (4-5)]
[561-577 (3-4)]
[561-579 (3-4)]
[561-588 (3-5)]
[563-588 (3-5)]
[589-595 (1)]
[589-596 (1-2)]
[589-597 (1-2)]
[597-602 (1)]
[597-610 (2-3)]
[598-605 (2)]
[598-606 (2)]
[598-610 (2-3)]
[601-610 (2-3)]
[602-610 (2-3)]
[603-609 (2)]
[603-610 (2)]
[604-610 (2)]
[605-610 (1-2)]
[606-610 (1)]
[611-616 (1)]
[622-626 (1)]
[623-626 (1)]
[624-634 (2)]
[625-632 (2)]
[625-634 (2)]
[627-632 (1-2)]
[627-634 (2)]
[635-647 (2-3)]
[635-648 (2-3)]
[636-646 (2-3)]
[639-646 (1-2)]
apo-4C
Na-4C
Ca-4C
apo-21C
Na-21C
Ca-21C
%D
log time [s]

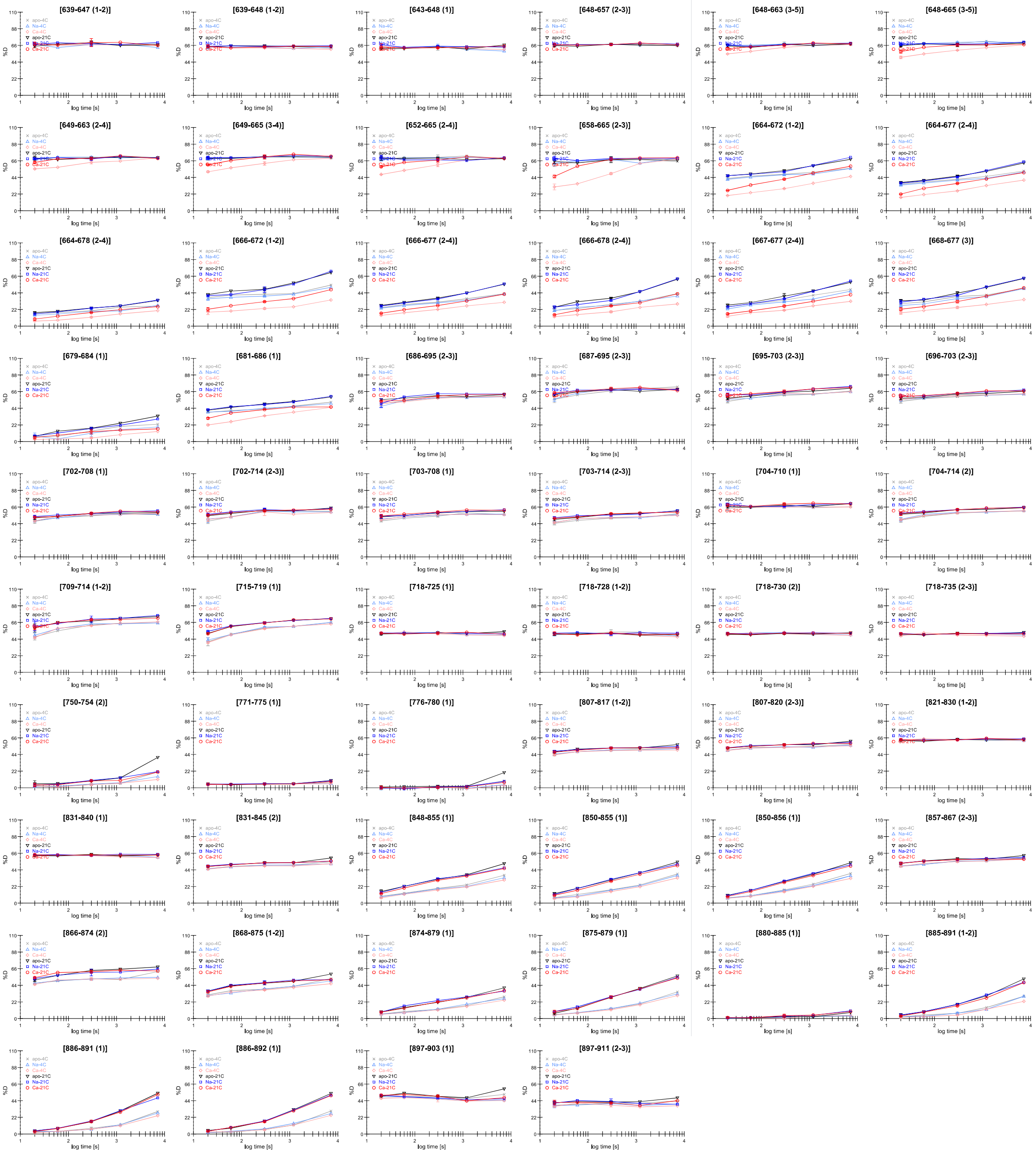

Supplementary Figure 2. Deuterium uptake plots in HDX-MS experiments. The deuterium uptake at the indicated time points and experimental conditions for each peptide.

Supplementary Table 1. Molecular dynamics simulations system setup.

	NCX1.4 apo	NCX1.4 Ca^{2+}-bound
Number of simulations	3	3
Simulation box dimensions (Å)	99 x164 x 189	99 x 164 x 189
Total number of atoms	312106	312400
Number of water molecules	78857	78957
Salt concentration (KCl)	0.15 M	0.15 M
Lipid composition – type	POPC	POPC
Lipid composition – number	455	455
