# Supplementary material for: Structural dynamics of Na+ and Ca2+ interactions with full-size mammalian NCX
Source: Commun Biol. 2024 Apr 16;7:463. doi: 10.1038/s42003-024-06159-9 (PMC11021524; doi:10.1038/s42003-024-06159-9)
Supplement: Supplementary file 2 — Description of Additional Supplementary Files [file 42003_2024_6159_MOESM2_ESM.pdf]

Description of Additional Supplementary Files

File name: Supplementary Data 1

Description: The source data behind Figures 1C, 1D, and 5.

File name: Supplementary Data 2

Description: PDB files of the initial and final coordinates of the MD simulations.
